# Supplementary material for: Remote measurement based care (RMBC) interventions for mental health—Protocol of a systematic review and meta-analysis
Source: PLoS One. 2024 Feb 16;19(2):e0297929. doi: 10.1371/journal.pone.0297929 (PMC10871474; doi:10.1371/journal.pone.0297929)
Supplement: S2 Table — (DOCX) [file pone.0297929.s002.docx]

**S2 Table.** **Inclusion and exclusion criteria related to the PICOS design**

| **PICOS domain** | **Inclusion criteria** | **Exclusion criteria** |
| --- | --- | --- |
| P | Participants were diagnosed with a mental health disorders defined by DSM-V or ICD-10 F-diagnoses or Z73 (Problems related to life-management difficulty) | No formal psychiatric diagnosis |
|  | Adults |  |
|  |  | Intervention delivered to family members (either as the target recipients of the intervention or in addition to the patients) |
| I | Remote mental health symptom tracking | No remote mental health symptom tracking (including psychiatric symptom only tracking immediately before, after, or during a clinical encounter) |
|  | Report of self-reported (individual) experience | No report of individual experience (including psychiatric symptom tracking only through passive sensing / monitoring) |
| C | Not specified | Not specified |
| O | Quantitative data |  |
|  | Symptom-focused outcomes |  |
|  | Relapse |  |
|  | Recovery-focused outcomes (e.g. empowerment, self-efficacy, hope, social connectedness) |  |
|  | (Global) functioning |  |
|  | Quality of life |  |
| S | Longitudinal observational study designs, including retrospective and prospective studies |  |
|  | Experimental study designs, including randomised controlled trials and cluster randomised control trials | Theoretical or statistical models |
|  | Mixed methods study designs | Systematic reviews and meta analyses |
|  | Feasibility or pilot study designs | Case studies |
|  | Manuscript written in English or German language | No original research contribution (letters to the editors, opinions) |
