# Supplementary material for: Remote measurement based care (RMBC) interventions for mental health—Protocol of a systematic review and meta-analysis
Source: PLoS One. 2024 Feb 16;19(2):e0297929. doi: 10.1371/journal.pone.0297929 (PMC10871474; doi:10.1371/journal.pone.0297929)
Supplement: S3 Table — (DOCX) [file pone.0297929.s003.docx]

**S3 Table. Search syntax, date of searches, and number of results returned for each database, number of references for review**

| **Database** | **Date of search** | **Results** | **Search syntax** |
| --- | --- | --- | --- |
| PubMed | 24.08.2022 | 1456 | ("Mental Disorders" [MeSH Terms] OR "Stress, Psychological" [MeSH Terms]) AND ("Self Report" [MeSH Terms] OR "patient reported outcome measures" [MeSH Terms] OR "ecological momentary assessment" [MeSH Terms] OR ("symptom*"[All Fields] AND "monitor*"[All Fields]) OR ("symptom*"[All Fields] AND "assessment*"[All Fields]) OR ("patient"[All Fields] AND "monitor*"[All Fields]) OR ("remote*"[All Fields] AND "monitor*"[All Fields]) OR ("remote*"[All Fields] AND "assessment*"[All Fields]) OR ("remote*"[All Fields] AND "symptom*" [All Fields] AND "monitor*"[All Fields]) OR ("remote*"[All Fields] AND "symptom*" [All Fields] AND "assessment*"[All Fields]) OR ("remote*"[All Fields] AND "patient"[All Fields] AND "monitor*"[All Fields]) OR ("measurement*"[All Fields] AND "based"[All Fields] AND "care"[All Fields]) OR ("ambulator*"[All Fields] AND "assessment*"[All Fields]) OR ("electronic*"[All Fields] AND "diar*"[All Fields]) OR ("personal*" [All Fields] AND "diar*"[All Fields])) AND ("digital technology"[MeSH Terms] OR ("mobile"[All Fields] AND "application*"[All Fields]) OR "telemedicine"[MeSH Terms] OR "smartphone"[MeSH Terms] OR "cell phone"[MeSH Terms] OR "text messaging"[MeSH Terms] OR "electronic mail"[MeSH Terms] OR "internet based intervention"[MeSH Terms] OR "sms"[All Fields] OR "short message service" [All Fields] OR ("e-mental"[All Fields] AND "health"[MeSH Terms]) OR ("digital*"[All Fields] AND "mental health"[MeSH Terms]) OR ("technology"[MeSH Terms] AND "based"[All Fields] AND "intervention*"[All Fields])) |
| Medline | 24.08.2023 | 299 | 1. Mental Disorders/  2. Stress, Psychological/  3. 1 or 2  4. Self Report/  5. Patient Reported Outcome Measures/  6. Ecological Momentary Assessment/  7. (symptom* and monitor*).mp.  8. (symptom* and assessment*).mp.  9. (patient* and monitor*).mp.  10. (remote* and monitor*).mp.  11. (remote* and assessment*).mp.  12. (remote* and symptom* and monitor*).mp.  13. (remote* and symptom* and assessment*).mp.  14. (remote* and patient* and monitor*).mp.  15. (measurement* and based and care).mp.  16. (ambulator* and assessment*).mp.  17. (electronic* and diar*).mp.  18. (personal* and diar*).mp.  19. 4 or 5 or 6 or 7 or 8 or 9 or 10 or 11 or 12 or 13 or 14 or 15 or 16 or 17 or 18  20. Digital Technology/  21. (mobile and application*).mp.  22. Telemedicine/  23. Smartphone/  24. Cell Phone/  25. Text Messaging/  26. Electronic Mail/  27. Internet-Based Intervention/  28. sms.mp.  29. (short and message and service).mp.  30. (e-mental and health).mp.  31. (digital and mental and health).mp.  32. (technology and based and intervention*).mp.  33. 20 or 21 or 22 or 23 or 24 or 25 or 26 or 27 or 28 or 29 or 30 or 31 or 32  34. 3 and 19 and 33 |
| Embase | 24.08.2024 | 620 | 1. Mental Disorders/  2. Stress, Psychological/  3. 1 or 2  4. Self Report/  5. Patient Reported Outcome Measures/  6. Ecological Momentary Assessment/  7. (symptom* and monitor*).mp.  8. (symptom* and assessment*).mp.  9. (patient* and monitor*).mp.  10. (remote* and monitor*).mp.  11. (remote* and assessment*).mp.  12. (remote* and symptom* and monitor*).mp.  13. (remote* and symptom* and assessment*).mp.  14. (remote* and patient* and monitor*).mp.  15. (measurement* and based and care).mp.  16. (ambulator* and assessment*).mp.  17. (electronic* and diar*).mp.  18. (personal* and diar*).mp.  19. 4 or 5 or 6 or 7 or 8 or 9 or 10 or 11 or 12 or 13 or 14 or 15 or 16 or 17 or 18  20. Digital Technology/  21. (mobile and application*).mp.  22. Telemedicine/  23. Smartphone/  24. Cell Phone/  25. Text Messaging/  26. Electronic Mail/  27. Internet-Based Intervention/  28. sms.mp.  29. (short and message and service).mp.  30. (e-mental and health).mp.  31. (digital and mental and health).mp.  32. (technology and based and intervention*).mp.  33. 20 or 21 or 22 or 23 or 24 or 25 or 26 or 27 or 28 or 29 or 30 or 31 or 32  34. 3 and 19 and 33 |
| PsycINFO | 24.08.2025 | 523 | (Mental Disorder* OR psychological stress) AND (Self Report OR patient reported outcome measures OR ecological momentary assessment OR (symptom* AND monitor*) OR (symptom* AND assessment*) OR (patient AND monitor*) OR (remote* AND monitor*) OR (remote* AND assessment*) OR (remote* AND symptom* AND monitor*) OR (remote* AND symptom* AND assessment*) OR (remote* AND patient AND monitor*) OR (measurement* AND based AND care) OR (ambulator* AND assessment*) OR (electronic* AND diar*) OR (personal* AND diar*))  AND (digital technology OR (mobile AND application*) OR telemedicine OR smartphone OR cell phone OR text messaging OR electronic mail OR internet based intervention* OR sms OR short message service" OR (e-mental AND health) OR (digital* AND mental health) OR (technology AND based AND intervention*)) |
| **Total references** | | 2898 |  |
| **References for review** | | 2314 | After removal of duplicates |
