# Supplementary material for: Remote measurement based care (RMBC) interventions for mental health—Protocol of a systematic review and meta-analysis
Source: PLoS One. 2024 Feb 16;19(2):e0297929. doi: 10.1371/journal.pone.0297929 (PMC10871474; doi:10.1371/journal.pone.0297929)
Supplement: Attachment — Submitted filename: Rebuttal letter.docx [file pone.0297929.s004.docx]

PLOS One

Carlyle House
Carlyle Road
Cambridge, CB4 3DN
United Kingdom

April 8th, 2023

Rebuttal Letter

Dear Mr. Demurtas,

Thank you for taking the time to review our protocol titled Remote measurement based care (RMBC) interventions for mental health - Protocol of a systematic review and meta-analysis. We appreciate the effort the editorial team and the reviewers have put into providing feedback on our work. We have taken your comments into careful consideration and have made the necessary revisions:

- According to one of the reviewers' comments, we have reworked the language of our protocol and improved its readability.
- Furthermore we have edited the manuscript so it meets PLOS ONE's style requirements, updated the financial disclosure and competing interests section.

Thank you once again for your valuable feedback and I look forward to hearing back from you.

Sincerely,

Dr. Felix Machleid
